# Mineral precipitation-induced porosity reduction and its effect on transport parameters in diffusion-controlled porous media

**DOI:** 10.1186/s12932-015-0027-z

**Published:** 2015-09-03

**Authors:** Aurélie Chagneau, Francis Claret, Frieder Enzmann, Michael Kersten, Stephanie Heck, Benoît Madé, Thorsten Schäfer

**Affiliations:** Institute for Nuclear Waste Disposal (INE), Karlsruhe Institute of Technology (KIT), Karlsruhe, Germany; Department of Earth Sciences, Freie Universität Berlin, 12249 Berlin, Germany; Department of Geosciences, Johannes Gutenberg University, Mainz, Germany; Water and Environment Division, French Geological Survey (BRGM), Orléans, France; French Agency for Nuclear Wastes Management (ANDRA), Châtenay-Malabry, France

**Keywords:** Mineral precipitation, Reactive transport, Porous media, Through diffusion, CT, Archie’s law, Model

## Abstract

**Background:**

In geochemically perturbed systems where porewater and mineral assemblages are unequilibrated the processes of mineral precipitation and dissolution may change important transport properties such as porosity and pore diffusion coefficients. These reactions might alter the sealing capabilities of the rock by complete pore-scale precipitation (cementation) of the system or by opening new migration pathways through mineral dissolution. In actual 1D continuum reactive transport codes the coupling of transport and porosity is generally accomplished through the empirical Archie’s law. There is very little reported data on systems with changing porosity under well controlled conditions to constrain model input parameters. In this study celestite (SrSO_4_) was precipitated in the pore space of a compacted sand column under diffusion controlled conditions and the effect on the fluid migration properties was investigated by means of three complementary experimental approaches: (1) tritiated water (HTO) tracer through diffusion, (2) computed micro-tomography (µ-CT) imaging and (3) post-mortem analysis of the precipitate (selective dissolution, SEM/EDX).

**Results:**

The through-diffusion experiments reached steady state after 15 days, at which point celestite precipitation ceased and the non-reactive HTO flux became constant. The pore space in the precipitation zone remained fully connected using a 6 µm µ-CT spatial resolution with 25 % porosity reduction in the approx. 0.35 mm thick dense precipitation zone. The porosity and transport parameters prior to pore-scale precipitation were in good agreement with a porosity of 0.42 ± 0.09 (HTO) and 0.40 ± 0.03 (µ-CT), as was the mass of SrSO_4_ precipitate estimated by µ-CT at 25 ± 5 mg and selective dissolution 21.7 ± 0.4 mg, respectively. However, using this data as input parameters the 1D single continuum reactive transport model was not able to accurately reproduce both the celestite precipitation front and the remaining connected porosity. The model assumed there was a direct linkage of porosity to the effective diffusivity using only one cementation value over the whole porosity range of the system investigated.

**Conclusions:**

The 1D single continuous model either underestimated the remaining connected porosity in the precipitation zone, or overestimated the amount of precipitate. These findings support the need to implement a modified, extended Archie’s law to the reactive transport model and show that pore-scale precipitation transforms a system (following Archie’s simple power law with only micropores present) towards a system similar to clays with micro- and nanoporosity.

**Electronic supplementary material:**

The online version of this article (doi:10.1186/s12932-015-0027-z) contains supplementary material, which is available to authorized users.

**Graphical abstract:**
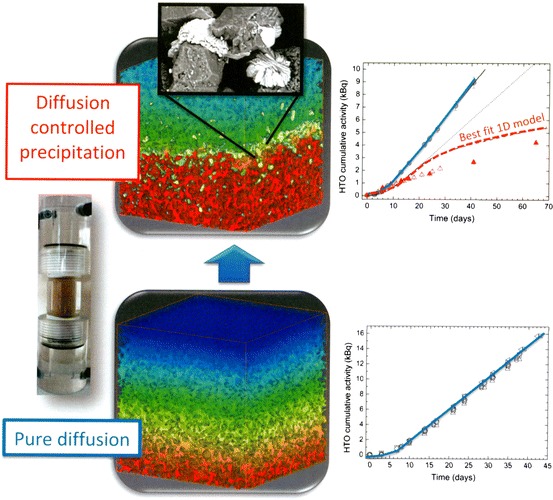
:

## Background

Understanding how changing porosity as a results of chemical reactions affects permeability and tortuosity is of paramount importance for describing various hydrogeological processes, such as fluid circulation in acid mine drainage remediation [1] or hydrothermal systems [2, 3], management of aquifer recharge [4], efficiency of permeable reactive barriers [5] and the long-term geochemical evolution of host rock considered for high-level nuclear waste repositories [6, 7]. In some cases a porosity and flow reduction (clogging) effect is desired and intended to inhibit fluid and solute migration (e.g. mitigation strategies for CO_2_ leakage along wellbores [8] or for microbial-enhanced-hydrocarbon-recovery [9]). Describing clogging phenomena in a reactive transport code is challenging [10], particularly over the long periods (1,000 to 1,000,000 years) that are of interest in most safety assessment cases. In addition to validating numerical schemes, experiments validating the laws implemented for coupling the geochemistry (e.g. dissolution-precipitation reactions) to the transport parameters (induced porosity changes) and overall fluid flow processes (e.g. Carman-Kozeny relationship or Archie’s law) are required.

Archie’s law [11] is widely used in reactive transport models [12]. It describes the ratio between the effective diffusivity of a dissolved species in a porous medium (D_e_, in m^2^ s^−1^) and the diffusion coefficient of that same species in pure water (D_w_, in m^2^ s^−1^) (Eq. 1). The D_e_/D_w_ ratio is a function of the tortuosity of the material (τ) and its constrictivity (δ) and therefore of the material’s accessible porosity (ε_a_) [13]. As both the tortuosity and the constrictivity are difficult to measure experimentally, their ratio is often described as the geometrical factor, G. The relationship can be written as follows:

1 $$\frac{{D_{e} }}{{D_{w} }} = \frac{\delta }{\tau } = \frac{1}{G} = \varepsilon_{a}^{m}$$

where *m* is a fitting factor often called the cementation exponent [14]. It has been shown in a compilation of clays and shales that the tritium D_e_ –ε_a_ relationship can be reasonably well described with a cementation factor ranging between 2 and 3 and the Cl^−^ D_e_ –ε_a_ relationship with a cementation factor between 2 and 2.5, respectively [15, 16]. However, the application of Archie’s Law to complex, heterogeneous systems has been shown to be inaccurate [12]. Another issue with the application of Archie’s Law to complex networks is how well it describes systems in which the porosity has been reduced or enhanced solely by chemical reactions. Work done by Tyagi, et al. on clay systems, where simulations on the effect of nanopores within particles (interlayer pores) and micropores between particles demonstrated that such systems can only be described by a sum of two power functions related to the micro- and nanoporosity [17]. Another study on gas permeability of reservoir rocks (Berea sandstone) have shown that two groups of rocks are differentiated according to the cementation factor *m*, again showing that the sandstone formation cannot be described properly using just one cementation factor [17].

In this study, we attempted to apply Archie’s law in a 1D single continuum reactive transport model for a simple sand system, reducing the pore space by pore scale precipitation. Numerical model inputs such as porosity, effective diffusion coefficient and the cementation factor applied in the Archie’s law have been derived from the experimental data obtained prior to the porosity reduction experiments. Afterwards, a systematic study of diffusion-controlled precipitation of a simple salt, celestite (SrSO_4_), in the poorly compacted sand was performed. Double-reservoir diffusion experiments are widely used in the literature to characterize the transport properties of porous materials [13, 18, 19] or in so called two-way U-tube experiments to control the precipitation of various mineral phases [20, 21] in porous silica gel. Our experiments presented here are a combination based on those two approaches mentioned above. The second method (3D imaging coupled to pore-morphology modeling) became of great interest in the past two decades in many domains, including CO_2_ sequestration [22, 23] and soil sciences [24], as a powerful and non-destructive analysis tool for characterizing a porous material and predicting how it will evolve under various petro-physical, geochemical, and hydrogeological conditions [25–28].

Our experimental approach consisted of three complementary techniques believed to characterize the reactive system in the most complete way: (1) conservative tracer experiments before and during the formation of a precipitation front, (2) computed micro-tomography (µ-CT) coupled to pore-morphology modeling before and after the formation of a precipitation front, and (3) post analysis of the precipitate (dissolution, SEM-EDX). The experimental data were used as input parameters for a 1D reactive transport model based on the CRUNCHFLOW code [29]. The calculated flux, mineral mass formed and porosity reduction were then compared to experimental results obtained after celestite precipitation.

## Results and discussion

### Initial transport parameters

**Fig. 1:**
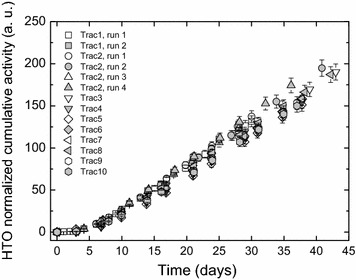
HTO through-diffusion curves obtained on 10 different diffusion cells (Trac1 to Trac10) in up to four experiments (or runs) performed on sea sand, prior to porosity reduction by precipitation. These are reported to give an idea of the data dispersion.

The initial transport parameters of the compacted sea sand were obtained on 10 independently packed sand columns, in up to four different through-diffusion experiments per column (n_tot_ = 16). The results as depicted in Fig. 1 show high reproducibility in the diffusion curves. The accessible porosity was 0.42 ± 0.09, and the effective diffusion coefficient D_e_ (4.48 ± 0.17) × 10^−10^ m^2^ s^−1^. The corresponding cementation factor calculated with Archie’s law was 1.92 ± 0.44. The geometric factor, calculated as the ratio of the diffusivity of HTO in pure water (2.24 × 10^−9^ m^2^ s^−1^) to the product of the effective porosity and diffusivity, was 2.11 ± 0.42.

The porosity obtained by the HTO tracing experiments (0.42 ± 0.09) was in good agreement with that obtained independently by Mercury Intrusion Porosimetry (MIP) (0.42 ± 0.02), and also with that based on the mass of sand introduced into the diffusion cells (0.42 ± 0.03). These values were also in good agreement with the values obtained by the pore morphology model on the segmented µ-CT volumes of six different diffusion columns (0.40 ± 0.03). The average pore radius obtained by both MIP (40 ± 2 µm) and the pore morphology model (50 ± 5 µm) also compared fairly well. The data for the initial sea sand transport parameters are summarized in Table 1.

**Table 1:** Summary of the average initial transport parameters of compacted sea sand obtained from three analytical techniques: MIP, tracing experiments and µCT

Technique	ε_a_	D_e_ ×10^−10^ m^2^ s^−1^	G
MIP	0.42 ± 0.02	–	–
HTO	0.42 ± 0.09	4.48 ± 0.17	2.11 ± 0.42
µCT	0.40 ± 0.03	2.21 ± 0.43	2.72 ± 0.39

### Evolution of transport parameters during porosity reduction (clogging)

**Fig. 2:**
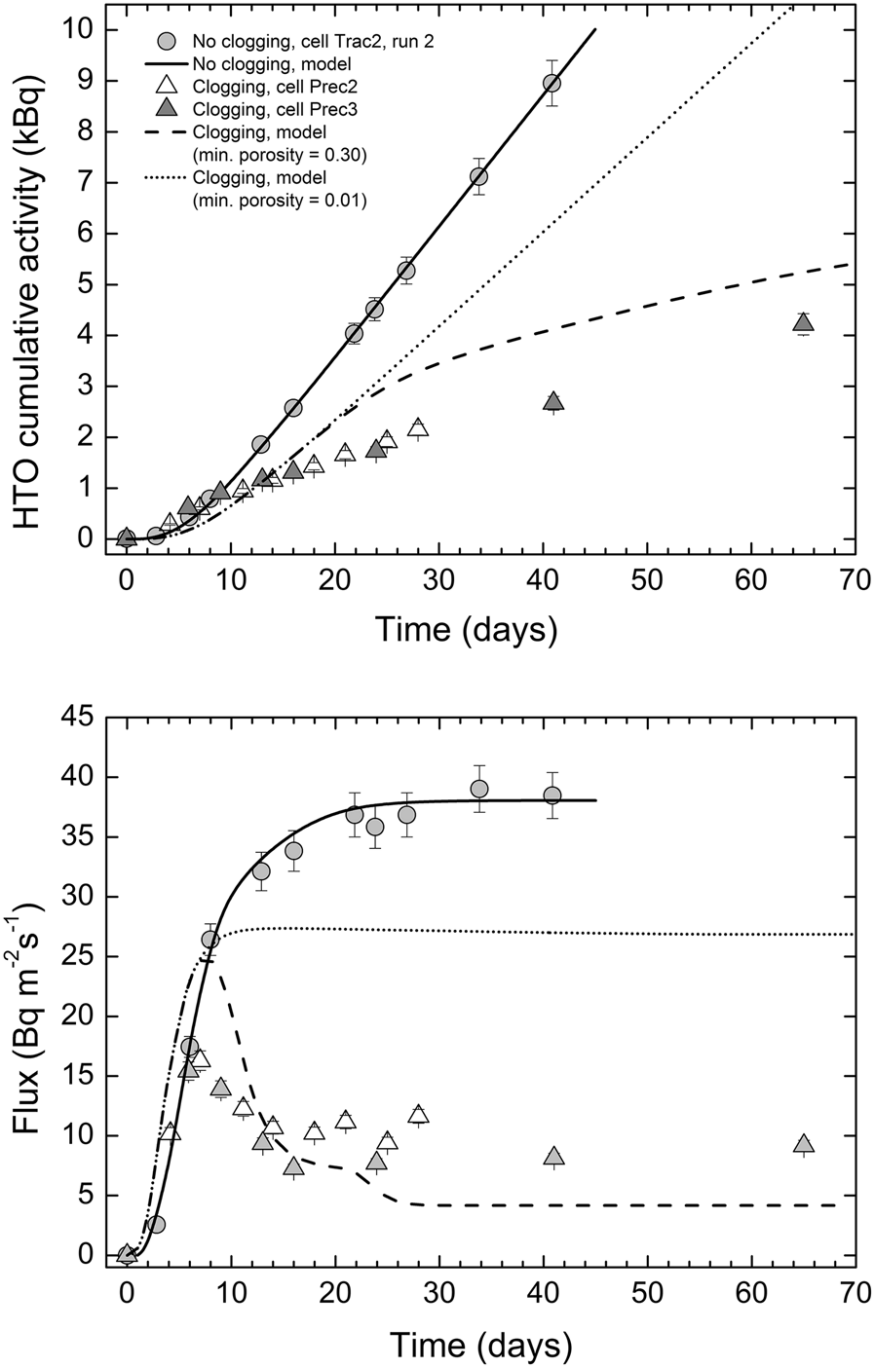
HTO through-diffusion (*upper graph*) and flux (*lower graph*) curves for cell Trac2, run 2 (*circles*) without pore space clogging (or reduction), and cells Prec2 and Prec3 (*open* and *full triangles*, respectively) while reducing porosity by celestite precipitation. The full line is the model without clogging (reduction). The *dashed* and *dotted lines* are obtained by running a reactive transport model taking into account the clogging (reduction) (see text for details), with a minimum porosity of 0.01 and 0.30, respectively.

The diffusion of the conservative tracer (HTO) in experimental cells Prec2 and Prec3 indicated the onset of porosity reduction (i.e., change in flux curve slope, Fig. 2), and the significantly decreased flux indicated a change in the material`s transport properties. Although tritium may substitute for hydrogen in water on clays and other hydrated mineral phases this reaction is not important relative to the mobility of tritium based on published laboratory and field studies [30]. All field studies reviewed in [30] indicate that tritium migrates at the same velocity as surface- and ground waters and this is also to be expected in this study with a non-hydrated mineral phase used as precipitate.

The precipitation front that was visible after 7 days to the naked eye formed a sharp disk located near the middle of the diffusion columns, slightly closer to the Sr reservoir. They appeared as white disks less than 1 mm thick, perpendicular to the direction of diffusion. The exact position was 21 ± 1 and 18 ± 2.5 mm from the filter supports of the Sr-reservoir for Prec1 and Prec3, respectively. The front in diffusion cell Prec1 was imaged with µ-CT at two different resolutions (5.89 and 15.2 µm), and the 3D volume was implemented in a pore-morphology model to estimate its influence on the initial transport parameters (porosity, diffusivity, and tortuosity). The Laplace field calculations consist of applying a non-reactive particle concentration gradient over the porous material and running a random-walk simulation, tracing the particles over a selected running time.

We saw the effect of porosity reduction on the tracer diffusivity in diffusion cells Prec2 and Prec3 after about 7 days of reaction (Fig. 2). The HTO flux while the pore space was being reduced by celestite precipitation significantly decreased compared with the breakthrough curves obtained in the materials’ initial state. The HTO flux increased during the first 6 days in a fashion similar to the behavior in the unreacted media. Afterwards, it decreased quickly until it reached steady state at a lower level at approximately day 15. The steady state flux had a value of 8 ± 1 Bq m^−2^ s^−1^, about 4.6 times lower than the fluxes obtained for the control (no precipitation) experiments (37 ± 2 Bq m^−2^ s^−1^). The fact that there was measurable flux even after 66 days of celestite precipitation indicates that at least part of the pore space in the reaction zone remained open and connected.

### Characterizing the precipitate

**Fig. 3:**
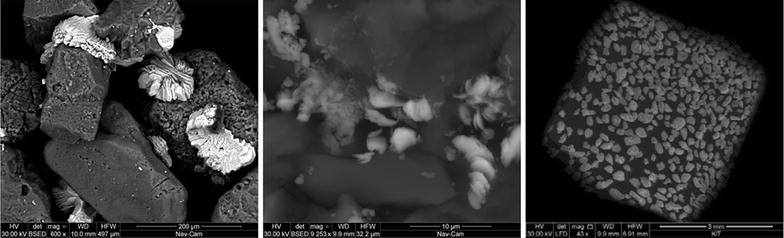
SEM back scattering (BS) images of the sand grains (*dark grey values*) and celestite aggregate grains (*light grey values*) in the precipitation zone, before (*left* and *middle*), and after (*right*) selective celestite dissolution (see text for details). In the latter case no celestite can be detected.

After preparing the samples for post-mortem analysis, and before the precipitate was dissolved, small samples from the precipitation zones in cells Prec2 and Prec3 were analyzed by SEM. Representative SEM backscatter images are displayed in Fig. 3. The precipitate was found in the form of aggregates of numerous microscopic crystals of various shapes and sizes. Some of the aggregates formed piles of tabular crystals, while others immediately adjacent to these piles consisted of clusters of needle-shaped crystals. The precipitate appeared to form preferentially on the rough surface of the sand grains, filling the micro-cracks. The aggregates were found isolated on the grains’ surfaces, or filling the pore space between several grains, thus cementing them together. The size of the individual celestite crystals in the aggregates ranged from a few µm to more than 50 µm. A significant number of small crystals were found also as isolated “dust” on the surface of the sand grains, often observed in surface cavities or micro-cracks. An EDX analysis of some of the crystals confirmed that the precipitate was stoichiometric celestite (SrSO_4_). Strontianite (SrCO_3_) was not found, even though the experiments were performed under atmospheric conditions. The SrCO_3_ saturation index (SI = log_10_(IAP/K_sp_)) defined here as the ratio between the ion activity product (IAP) and the solubility product K_sp_ was estimated to be −0.42 under the experimental conditions for a mixture of 0.5 M SrCl_2_ and 0.5 M Na_2_SO_4_ solutions in equilibrium with atmospheric CO_2_.

### µCT imaging of a precipitation front

**Fig. 4:**
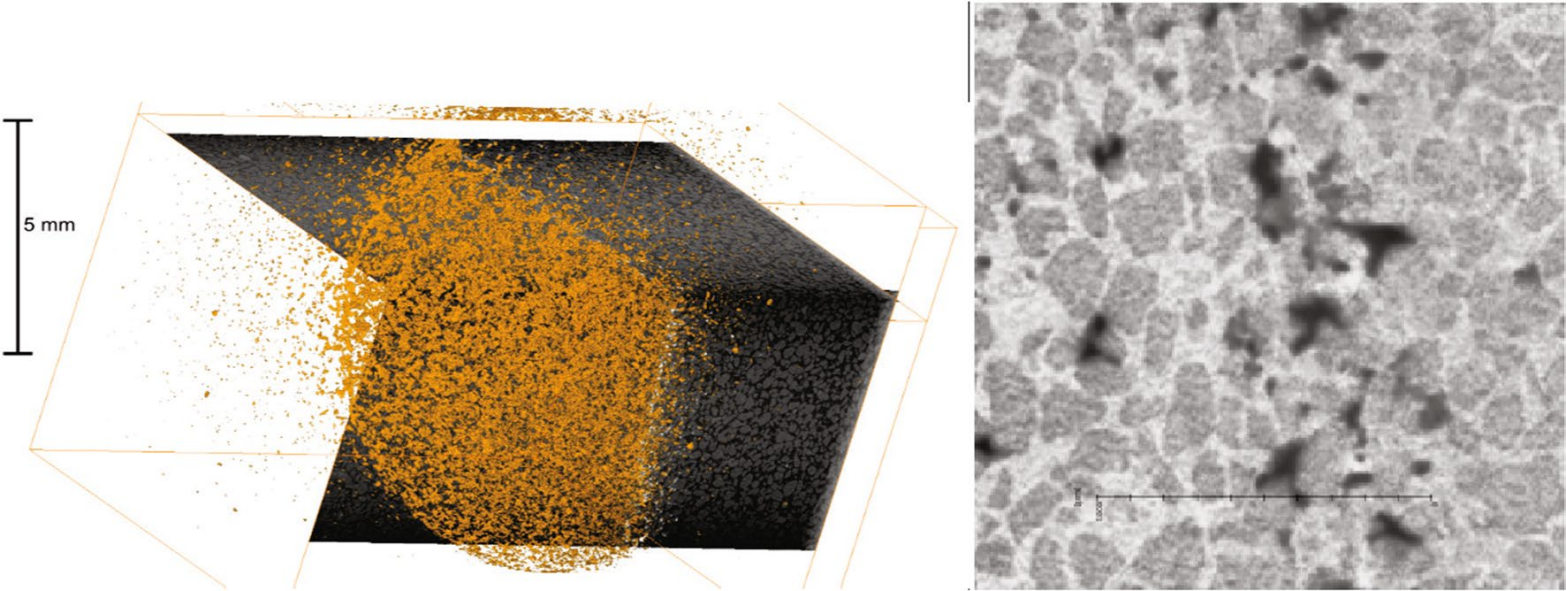
*Left* 3D rendering of the segmented celestite grains (in *orange*) from the high resolution CT images of experiment Prec1. *Right* celestite precipitation zone with celestite appearing in *dark grey*, sand grains in *light grey* and pore space in *white* (horizontal length 1.5 mm).

We mapped the precipitation front in diffusion cell Prec1 with µCT twice after 63 days of reaction, one at low resolution (15.1 µm) mapping the central 30 mm of the diffusion column, and one at higher resolution (5.9 µm) mapping the central 12 mm across the clogging area. Under both resolutions, the precipitation front could be clearly distinguished from the sand grains and water-filled pore space. The celestite grains appeared as bright white spots after reconstruction (Fig. 4), as celestite’s X-ray mass absorption coefficient is very high compared to the sand. The precipitation front was symmetrical and consisted of two distinct components: a dense precipitation disk about 0.35 mm thick surrounded on both sides by a homogeneously disperse precipitation zone about 3 mm wide. In the dense disk, the precipitates appeared to cement the sand grains together by filling the pores and some of the pore throats. The celestite did not fully cement the sand, as some pathways remained open, connecting one side of the front to the other. In the disperse precipitation zone, the precipitates were isolated and appeared to fill only the pores.

#### Porosity and precipitate mass quantification by low resolution µCT

We attempted to estimate the mass of precipitate in the pore space and the remaining porosity using a simple pixel-counting treatment. We estimated a volume of celestite of 5.1 ± 0.8 mm^3^ and a corresponding mass of 20.2 ± 3.2 mg. This mass is in good agreement with the dissolved volume of celestite in the 5 mm thick sample containing the precipitation front detected in the post mortem analysis of the same diffusion cell, which gave 21.7 ± 0.4 mg.

The initial porosity of the sand column was estimated with a similar pixel-counting approach for the pore space in the regions far from the precipitation zone. An initial porosity was estimated at 0.44 ± 0.05, again in reasonable agreement with all other experimental results (0.40–0.42). From the volume of precipitated celestite and the initial porosity, we were able to estimate the minimum porosity reached in the 350 µm thick dense precipitation zone. Considering that approximately 75 % of the precipitate was included in this dense precipitation zone, we estimated that the porosity in this area was ≥0.28 ± 0.03. Unfortunately, it was not possible to estimate the connectivity of this residual porosity with the low spatial resolution of the micro-tomography.

#### Porosity and precipitate mass quantification by high resolution µCT

The mass of celestite precipitate and the porosity profile were determined with simple voxel-counting on the segmented volume of the high-spatial resolution dataset. This method gave a mass of precipitate of 30.2 mg, a value higher than the estimate determined by dissolving the precipitate during post-mortem analysis of the same diffusion cell (21.7 ± 0.4 mg). This difference in values might be due to the over estimate of the gray values threshold that we selected for celestite prior to the beam hardening correction. A similar voxel counting directly on the measured images without filtering gave a value of 25 ± 5 mg. The discrepancy observed cannot be attributed to incomplete precipitate dissolution during the post mortem analysis, since SEM-EDX analysis showed no residual celestite after acid treatment (Fig. 3). The discrepancy might be due to precipitate microporosity, since a close look at the untreated CT images reveals that the precipitate has variable grey values, corresponding to possible variable densities (see Fig. 4). On the SEM images of the Prec2 and Prec3 precipitates, the aggregates appeared to show micro- or even nanoporosity in between the individual celestite crystals (Fig. 3).

**Fig. 5:**
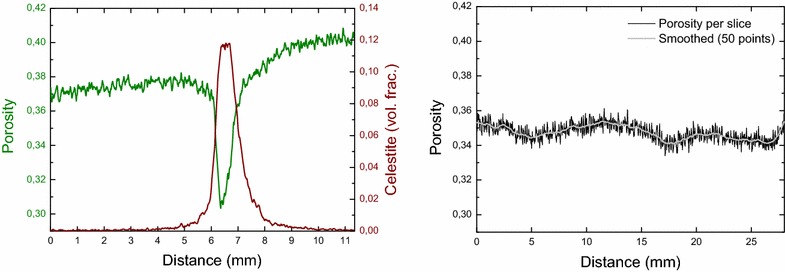
Precipitated celestite and porosity profile in diffusion cell Prec1 (*left*) and in a reference diffusion cell without celestite precipitation (*right*) using voxel-counting.

The shape of the celestite aggregates seems to indicate that the crystals have grown on the surface of the sand grains and filled the adjacent pores, leading to cementation of the porous material in the precipitation zone. However, this cementation did not completely fill the pore space (Fig. 4), and a significant portion of the pore space remained unfilled, as is suggested by the porosity profile obtained by the voxel-counting method on the segmented CT data set for Prec1 (Fig. 5). This observation is in good agreement with the steady-state through-diffusion HTO data observed after the formation of the precipitation front. The minimum porosity reached in this front estimation based on the voxel-counting method was 0.31 ± 0.03. This value is in good agreement with the value found for the low resolution data set (0.28 ± 0.03).

The small increase in porosity before and after the precipitation front observed on the porosity profile suggests variability in the sand compaction. This variability was also observed in a reference diffusion cell (in which no celestite precipitation occurred) measured with CT (Fig. 5). The variation in this reference cell was quasi-periodic, with an amplitude of ±0.03 for an average porosity of 0.35.

#### Transport parameter quantification by high resolution µCT

**Fig. 6:**
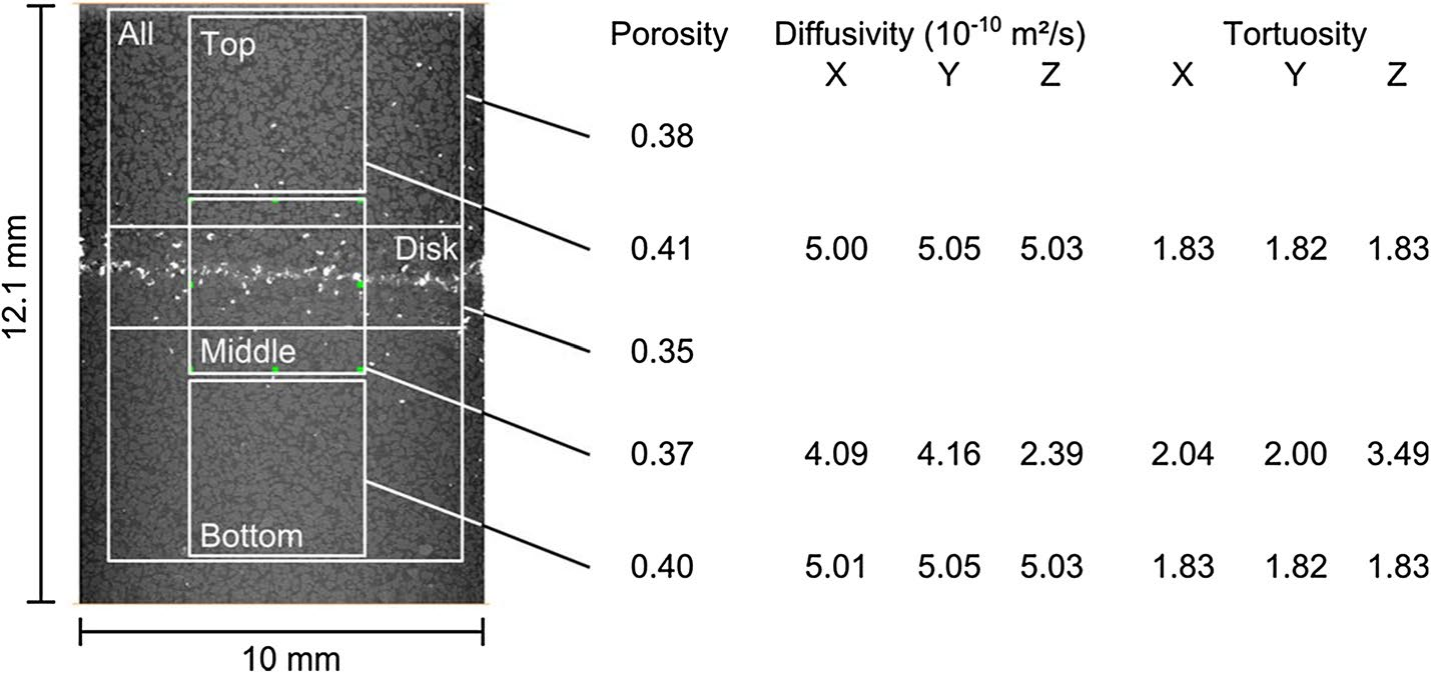
Schematic representation of the location of the different ROIs selected from the high resolution CT data set, and transport parameters calculated with GeoDICT for each ROI. The Z direction is the longitudinal axis, 12.1 mm long, whereas the X and Y axis are the lateral axis given by the diameter of the column (10 mm).

We implemented the segmented 3D volume with three labels (pore space, sand grains and precipitate) in the pore-morphology model to quantify the transport parameters in the undisturbed material and in the precipitation zone. Different Regions of Interest (ROIs) were selected from the complete data set (Fig. 6): (1) a cylinder cut-out from the center of the volume (ROI-all), excluding the borders to avoid rim effects (2) three cubes of the same dimensions (3.6 mm side length) at different positions in the volume, two of them in the undisturbed material above (ROI-top) and below (ROI-bottom) the precipitation zone, and one in the precipitation zone (ROI-middle); and (3) a disk cut-out from the center of the volume (ROI-disk) that included the precipitation zone.

The average porosity for the undisturbed porous material in the three Cartesian directions was calculated for ROI-top and ROI-bottom. The pore-morphology model interpreted neighboring voxels as belonging to the same connected group if they were in contact by either their face, edge or vertex. Isolated groups of voxels connected with the pore value were seen as isolated pores. The total porosity was 0.41 ± 0.01, with no isolated pores detected (Fig. 6). This value is in good agreement with the HTO diffusion experiments (0.42 ± 0.09), MIP (0.42 ± 0.02), the µ-CT calculations on the reference diffusion cells (0.40 ± 0.03) and the voxel counting approach on the low (0.44 ± 0.05) and high (0.39 ± 0.03) resolution data sets.

The average effective diffusivity in the three Cartesian directions was calculated with a bulk (Laplace) diffusion approach. This is a continuum mechanics approach using the Laplace equation in pores applying the Neumann boundary conditions at the pore-surface interface. D_e_ was found to be (5.03 ± 0.02) × 10^−10^ m^2^ s^−1^ for the ROI-top and ROI-bottom. This value is in good agreement with the HTO tracing experiments (4.48 ± 0.17) × 10^−10^ m^2^ s^−1^).

The average tortuosity factor of the undisturbed material in the three Cartesian directions was calculated for ROI-top and ROI-bottom using the ratio of the pore-morphology model derived porosity and effective diffusivity. It was 1.83 ± 0.02, which is again in good agreement with the geometric factor calculated based on HTO diffusion (1.92 ± 0.44).

In the precipitation zone, the porosity calculated was only slightly lower, as it was an average of the entire ROI-middle, which included portions of the unreacted material. However, the tortuosity increased and the diffusivity decreased significantly. The model indicated that no isolated pores occurred, suggesting that the porosity remaining in the precipitation zone is fully connected.

**Fig. 7:**
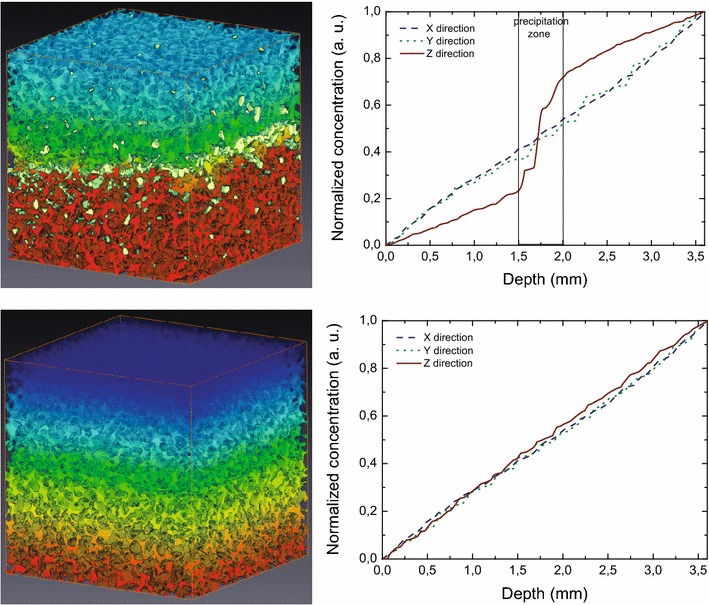
3D representation of the Laplace field in the Z direction (*right*) for the ROI middle (*top*) and ROI bottom (*bottom*), and corresponding graphic visualization (*left*). On the *top left* image, the celestite grains appear in *white.*

The influence of the increased tortuosity and decreased porosity in the precipitation zone on dissolved species transport can be better understood from the Laplace field calculations performed on the middle and bottom ROIs (Fig. 7). The coupled GeoDict software code (Math2Market) iteratively simulates fluid flow using the Navier–Stokes-Brinkman equation, and subsequently creates particle diffusion paths employing its AddiDict module. The Laplace field was performed in all three Cartesian directions for both ROI-middle and ROI-bottom. The field was very homogeneous in the X, Y and Z directions for the bottom ROI, as expected for a homogeneous, undisturbed porous material. It was also homogeneous in the X and Y directions for the middle ROI, but was strongly influenced by the precipitation zone in the Z direction, the direction of diffusion from Sr^2+^ to the SO_4_^2−^-reservoir.

**Fig. 8:**
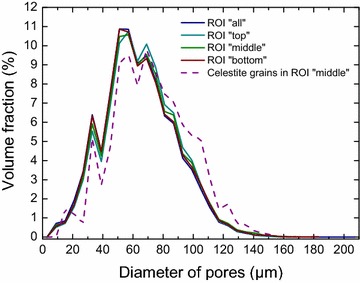
Geometric pore size distribution and celestite grain size distribution calculated with the pore morphology model for different ROIs.

The geometrical pore size distribution was calculated by fitting spheres of increasing radius in the pores. The centers of the spheres were placed in the center of the pores, and their radii increased with a step size of 1 voxel length (5.9 µm), until the spheres reached the surface of the sand and celestite grains. The final sphere radius corresponded to the pore radius. The distribution was very similar for all ROIs (Fig. 8). A similar calculation was made for the celestite grain size distribution. The distribution curve was the same as for the material’s pores, indicating that the material fills the pore space, in agreement with the SEM observations of the precipitate. The small shift towards larger diameters indicates that the precipitate may have developed preferentially in the largest pores, an observation made in compacted illite [31].

### Modeling

Prior to reactive transport modeling, the CRUNCHFLOW model was calibrated on the pure HTO-diffusion experiments in which no precipitation occurred. The model was run for each independent experiment, with a mesh size of 0.1 mm. The calculated diffusion curves fit the experimental data well (Fig. 2, solid line) and allowed us to determine the effective diffusion coefficient of HTO, the porosity of the porous media and the cementation factor *m* in Archie’s Law.

The celestite precipitation rate was then introduced to the model, and an extensive parameter sensitivity study was carried out in which the kinetic regime, mesh size and celestite surface area were varied (see Additional file 1). It was shown that the results of the model depended on mesh size when larger than 0.1 mm, as previously reported [32, 33]. Therefore, the mesh size was limited to 0.1 mm for all further calculations (Additional file 1: Figure S1 and Figure S2). The modeled diffusion and flux curves, mass of precipitate, and porosity profiles were only slightly dependent on the celestite’s kinetic regime (5.13 × 10^−8^ mol m^−2^ s^−1^ for kinetics or 1.0 × 10^−3^ mol m^−2^ s^−1^ for quasi-equilibrium). Furthermore, published values for celestite surface area vary between 0.44 and 72.7 m^2^ g^−1^ (e.g. [34, 35]). In addition, two commercially available powders were measured with N_2_-BET and were found to have surface areas of 0.525 and 0.998 m^2^ g^−1^, respectively. The influence on model predictions was tested with the smallest (0.44 m^2^ g^−1^), the average (40 m^2^ g^−1^) and the largest (72.7 m^2^ g^−1^) surface area values. Results are reported in Fig. S3 of the Additional file 1. The model is only slightly dependent on the celestite surface area. The curves for 40 and 72.7 m^2^ g^−1^ are very similar. The curve for the 0.44 m^2^ g^−1^ case is quite different, as the peak is broader: the precipitation takes about 5 additional days to influence the flux drop. However, the minimum flux value is reached very shortly after the other cases, and the flux value reached is also very similar, as is the specific surface area (see Additional file 1). The main calibration parameters left were the minimum porosity and cementation factor. The minimum porosity is a value that can be set in the model input, such that further precipitation would produce no further porosity reduction.

Sr retardation due to sorption on the sand surface was neglected, as no significant sorption was observed experimentally in the batch sorption experiments under the conditions investigated as listed in Table 2. This observation is consistent with published data of Sr sorption on quartz [36], where no uptake could be found under the experimental condition (10^−5^ M Sr(II), solid concentration 100 g L^−1^, pH 5.5). Furthermore, taking published results on Langmuir-type adsorption of Sr(II) on kaolinite with a maximum uptake of 17.7 µmol g^−1^ found by Parkman et al. [37] and the kaolinite impurities in the sea sand of 2.25 wt% calculated based on the Al_2_O_3_ XRF analysis (see Table 3) a potential Sr sorption by kaolinite will only amount to ~0.02 % of the 0.5 M reservoir in the diffusion experiments. This amount of Sr is within the analytical uncertainty of the Sr analysis by ICP-OES and can be neglected. Sr sorption onto newly formed celestite can also be neglected based on published data [38].

**Table 2:** Summary of the initial experimental conditions for the Sr^2+^ sorption experiments on purified sea sand

Run	Contact time (h)	S/L ratio (g/L)	[NaCl] (mmol/L)	[Sr]_initial_ (mmol/L)	pH
1	36	10	1	0.001–1	5.5
2	24	25	1	0.05–0.1	5.5
3	24	50	1	0.05–0.1	5.5
4	24	100	1	0.05–0.1	5.5
5	24	150	100	0.05– 0.1	5.5
6	24	100	100	0.05	2–11.2
7	24	100	1	0.05	2–11.2
8	24	50	1	0.05–0.09	10
9	24	100	1	0.05 to 0.09	10

**Table 3:** XRF analysis of the raw and cleaned sand

	Major elements (wt%)					
	SiO_2_	Al_2_O_3_	K_2_O	TiO_2_	Fe_2_O_3_	CaO
Raw sand	99.1	0.88	0.42	0.10	0.09	0.03
Cleaned sand	99.2	0.89	0.42	0.09	0.09	0.03

	Minor elements (ppm)					
	Ba	Zr	Sr	Rb	Cr	Pb
Raw sand	105	64	19	14	7	3
Cleaned sand	102	55	18	13	7	4

The data from the HTO through-diffusion experiment using samples Prec2 and Prec3 where porosity reduction occurred, were used to evaluate the capability of the 1D single continuum model to reproduce the experimental data. More specifically, the porosity reduction linked to the precipitated amount of celestite and the HTO flux which is directly linked to this porosity reduction was modelled. Both experiments showed very similar HTO diffusion curves and mass of precipitate as determined by selective extraction (31.6 ± 0.4 mg for Prec2 and 35.6 ± 0.4 mg for Perc3); Table 4. The values for the initial porosity (0.42) and cementation factor (1.92) used were the average of all values obtained with HTO tracing experiments. The minimum porosity was used as a free-fit parameter for the modelling. Initially, a minimum porosity value of 0.30 was used, based on the µCT observations (Fig. 5). However, the HTO flux and diffusion curves modeled using this parameter setting deviated markedly from the experimental observations, clearly overestimating the final HTO flux (Fig. 2, dotted line) and showing behavior rather similar to the unclogged material. The best fit was obtained for a minimum porosity as low as 0.01 (Fig. 2, dashed line). However, for this value of minimum porosity, the mass of celestite precipitate in the pore space was unrealistically high with 148.8 mg after 80 days. This amount was about 4.5 times higher than the experimental value.

**Table 4:** Conditions of through-diffusion experiments in cells Prec1, Prec2 and Prec3 (clogging by celestite precipitation from 0.5 M SrCl_2_ and Na_2_SO_4_ solutions in reservoirs) and celestite masses determined

Experiment	Background (mol L^−1^)		HTO tracer (kBq L^−1^)	Duration of run (days)	Acid selective dissolution (mg)	µCT mass estimation (mg)
	NaCl	Si^4+^				
Prec1	0.001	None	None	362	21.7 ± 0.4	20–30*
Prec2	0.500	None	4,210	158	31.6 ± 0.4	nd
Prec3	0.500	1.74 × 10^−4^	4,240	78	35.6 ± 0.4	nd

High resolution: voxel counting raw data: 25 ± 5 mg; voxel counting after segmentation: 30.2 mg.

*nd* Not determined.

* Low resolution: 20.2 ± 3.2 mg.

Correspondingly, the final flux predicted by the model of 4.17 Bq m^−2^ s^−1^ is about two times lower than the experimentally determined steady-state flux after precipitation of 8.3 ± 0.9 Bq m^−2^ s^−1^ observed.

The use of a 1D single continuum model is controversial in reactive and highly heterogeneous media [23], even in simple systems such as the sea sand presented here. Molins et al. [23] have shown an overestimation of the dissolution in continuum-scale model simulations by a factor of ~1.9 for well-defined calcite dissolution experiments under advective transport conditions. Similarly, Tartakovski, et al. [39] have demonstrated that conventional low-resolution advective-dispersion equations produced erroneous results. By an adaptive high-resolution model based on homogeneous and heterogeneous reaction terms in Darcy-scale advection dispersion equations with grid sizes in the mixing zone smaller than the size of the sand grains, these authors were able to qualitatively reproduce the essential features of their experiment. These terms involve transport and mixing indices that account for highly non-uniform pore-scale concentration distributions and highly localized reactions and can serve as an alternative to computationally expensive high-resolution simulations.

Our reactive transport modeling of porosity reduction has shown that the exponential factor *m* in the Archie’s law formula, which regulates the transport response based on porosity reduction, has the strongest impact on the temporal and spatial evolution of the effective diffusion transport parameters affected by porosity reduction. Furthermore, the exponential factor *m* deviates strongly in different papers published on the same system as detailed below.

For example Shao et al. [40] used a cementation factor *m* of 2.0, while Steefel and Lichtner [41] used *m* = 1.0 to interpret the same data for the Maqarin natural analogue site. This suggests the need for extensions to the classical Archie’s law formulation concerning diffusive transport for systems evolving from high to low porosity as demonstrated here by mineral precipitation. First observations of the deviation from the Archie’s law were made by Ioannidis et al. [42] in porous media with porosity lower than about 0.2, where these authors observed a strong deviation of the formation factor on porosity. By fixing the microporosity between 0.38 and 0.23, theoretical work by Tyagi et al. [43] were able to show that the nanopores have an increasing effect on the overall transport parameters of the porous media.

## Conclusions

We have presented an approach of inter-diffusion of Sr and sulfate forcing supersaturation to describe mineral precipitation-induced porosity reduction in porous media under purely diffusion-controlled conditions. This approach is based on: (1) classical radiotracer inter-diffusion experiments involving Sr and sulfate driving supersaturation and precipitation of celestite; (2) µ-CT coupled to 3D pore-morphology modeling; and (3) post analysis consisting of selective dissolution and SEM-EDX data to quantitatively determine the amount of precipitated celestite. The combination of techniques gave consistent and complementary results.

This approach provided the experimental data needed to constrain reactive transport models for the diffusion–reaction system. Following our observations and analysis, our main conclusions are:

1. Celestite porosity reduction of sea sand was incomplete (the initial porosity was decreased by only 25 %), as evidenced by the non-zero steady-state HTO flux at the end of the experiment and the fact that micro-CT mapping indicates an open and connected porosity of 0.30. The celestite precipitate was primarily localized in a ~0.35 mm wide disk-shaped precipitation front surrounded by small areas of disperse precipitation in approximately the middle of the diffusion cell. The heterogeneity created by the precipitation front has a very strong influence on the transport parameters, despite the fact that the remaining porosity is fully connected. This phenomenon was evidenced by the strong effect of the porosity reduction on HTO flux and by the Laplace field calculation performed on the µ-CT 3D volumes.
2. The 1D single continuum reactive transport model successfully reproduced the experimental HTO through-diffusion data obtained prior to the sea sand porosity reduction with an average porosity of 0.40. However, the same model failed to reproduce the HTO through-diffusion during celestite precipitation, indicating that a single continuum model is not capable of describing the system. When the minimum porosity of 0.30 determined with µ-CT was used, the modeled tracer resembles more a homogeneously higher compacted system, which clearly contradicts the experimental observations. The best fit for the HTO through-diffusion flux curve was obtained using a minimum porosity of 0.01. However, the amount of precipitate obtained from this calculation was about 4.5 times greater than the experimental result. This can partly be explained because the reactive transport model was based on the assumption that all of the porosity (0.40–0.44) was available for precipitation. However, the simulated width of the zone affected by the precipitation was comparable with the experimental observations of diffusion cell Prec1.

From these observations we see that the system was well characterized, but that a simple 1D single continuum model cannot reproduce the pore reduction (clogging) experiments satisfactorily. A more complex dual continuum 2D/3D pore-scale reactive transport model including a modified diffusivity/porosity relationship and possibly complex precipitation kinetics (e.g. heterogeneous nucleation and inhibition processes) may provide a better description. Indeed, the SEM observations seemed to indicate an intricate mix between heterogeneous nucleation and surface growth processes, as was shown by the wide range of shapes and sizes of precipitated celestite. The question of why no more precipitation seems to occur in the column after a very short time (less than 10 days) should also be addressed in terms of inhibition processes.

This more complex model is needed to reproduce the experimental data presented here, and to predict reliably transport behavior in more complex, natural systems on long time scales, unreachable to the experimentalist.

## Experimental methods

We chose the porous material and diffusion cell’s dimensions based on several feasibility criteria; (1) the material grain size and pore diameters had to be large enough to be well resolved by µ-CT, but not so large as to allow for advective fluid flow, (2) the experiments had to run over a reasonable time period (1–2 months) (this limited the diffusion cell length) and (3) the cell geometry, material and wall thickness had to be suitable for the µ-CT equipment. The materials we chose and the diffusion cells are described in this section.

### Porous material characterization

The porous material used was the commercially available Merck purified sea sand with an average particle size of 100–300 µm (product no. 1.07711.5000). The sand was already purified by the manufacturer with acidic treatment and calcination. In another treatment step the sea sand was put in MilliQ water with a volume to mass ratio of 2:1. A small aliquot of 100 µL HNO_3_ was added to the suspension (pH ~3–4), which was then vigorously agitated for a few seconds. The turbid supernatant was poured out of the vessel a few seconds after agitation, giving enough time for the biggest particles to settle leaving the fines in suspension. This procedure was repeated 7-10 times until the supernatant appeared clear and a stable and a neutral pH was reached. The sand was then dried in an oven at 105 °C for at least 24 h (this sample is later referred as cleaned sand; Table 3). The N_2_-BET surface area of the powder was equal to 0.55 ± 0.02 m^2^ g^−1^. The mineralogical composition of the sand powder was characterized via XRD and XRF. The porosity and petro-physical properties of the compacted sand were determined with mercury intrusion porosimetry (MIP, before the formation of a precipitation front) and µ-CT (before and after the formation of a precipitation front).

#### Mineralogical characterization

X-ray diffraction (XRD) analysis on the bulk powder was performed on a Bruker D8 Advance equipped with a Cu K-alpha radiation source and a Sol-X energy dispersive detector. The analysis confirmed that the sand consists almost solely of silica (>99 % SiO_2_). The impurities were concentrated in order to be identified. For this purpose, a sample of the sea sand was suspended in MilliQ water and vigorously agitated for a few seconds. The largest particles settled within the first seconds following the agitation, but the supernatant remained turbid for about 3 h afterwards, due to the presence of numerous fine particles. A drop of the supernatant was sampled a few seconds after agitation and left to dry at room temperature on a XRD sample holder. The analysis of this sample revealed the presence of at least two minor mineral phases from the clay and mica groups.

The XRD analysis was confirmed by X-ray Fluorescence (XRF) measurements on the bulk powder (Table 3). The XRF were performed with a MagiXPRO, Fa. Philips, with a Rh anode at 3.6 kW. The trace elements were measured in undiluted powder pellets, and the major elements were measured at 3.2 kW on melt pellets diluted 14 times. The powder mainly consisted of silicon (97.8 wt% SiO_2_). Aluminum was identified as major impurity with 0.9 wt% Al_2_O_3_. Assuming the presence of Al solely in the form of kaolinite, this would correspond to 2.25 wt% kaolinite in the sea sand. The other major elements found were K, Ti, Fe and Ca, in decreasing order of abundance. As depicted from Table 3 the removal of fines from the raw material had no effect on the major element distribution.

Ba, Ce, Cr, Nd, Rb, Sr and Zr were present in trace amounts (10–100 ppm) and did not change during the purification step. Based on the Sr XRF analysis and the total mass of ~6.1 ± 0.3 g sea sand inserted in each column, the Sr amount was estimated to be 0.12 mg which would result, under the assumption of all Sr being bonded in celestite, in a total mass of 0.25 mg SrSO_4_ present in the total column. Such a small amount would not significantly influence the mass of precipitate obtained from SrSO_4_ dissolution.

#### Petrophysical properties and porosity of the compacted material

**Fig. 9:**
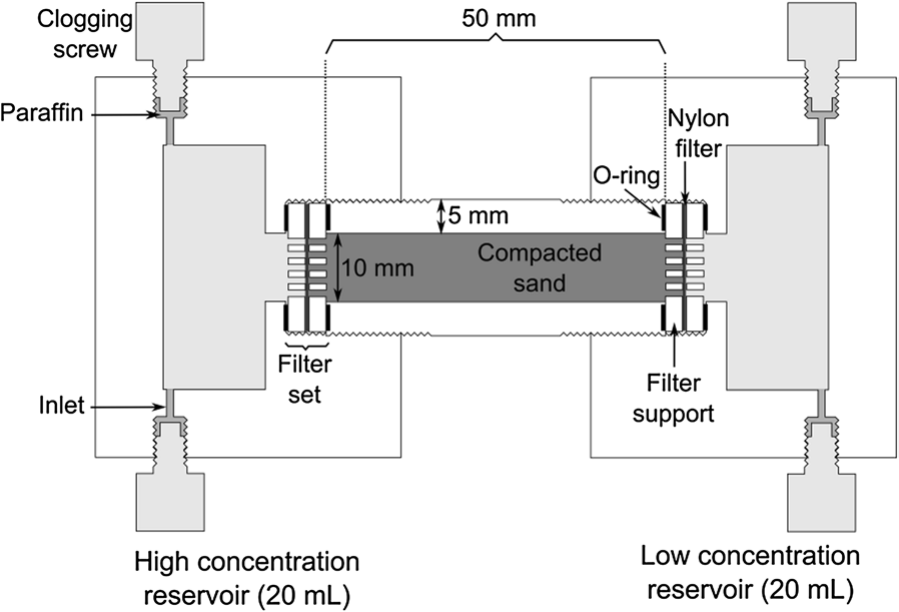
Schematic representation of a diffusion cell made of plexiglas showing a cross section with the dimensions. In the central part of the figure the column filled with sea sand (*dark grey*) is depicted and the high concentration reservoir spiked with tracer (*left side*) and the low concentration reservoir (*right side*) are shown.

The diffusion cells were specially designed to be suitable for µ-CT measurements. Construction material poly(methyl methacrylate) (C_5_O_2_H_8_)_n_ was chosen as it has very low X-Ray absorption in the energy range of 160–320 keV compared with the quartz-based porous material [44]. The columns were 50 mm long with 10 mm inner diameter and 5 mm wall thickness. The columns were filled with the porous material and horizontally fixed between two 20 mL capacitance reservoirs (Fig. 9). The column filling was separated from the reservoirs by 18 µm nylon filter gauze fixed between two pierced Plexiglas disks at both ends.

For the filling, the sand material was thoroughly cleaned with MilliQ water in order to remove impurities present as fine particles. To achieve this, the powder was suspended in twice its volume of MilliQ water and vigorously agitated. The turbid supernatant was discarded, and this procedure repeated until no visible turbidity was left in the supernatant. The sand grains were then dried in an oven at 105 °C for at least 24 h. After drying, the cleaned sand was equilibrated with a solution of either 1 mmol L^−1^ or 0.5 mol L^−1^ NaCl background electrolyte for a minimum of 3 days. The wet sand was scooped into the half-opened columns of the diffusion cells, and manually compacted by repeatedly tapping the cell onto the preparation table. Once the columns were filled and the supernatant water decanted, the cells were closed and the reservoirs were filled with the same background electrolyte as was used to saturate the pore space. An approximate porosity could be calculated for the material from the mass of sand compacted in the diffusion cells (about 6.1 ± 0.3 g) and the volume of the sand column (50 mm long by 10 mm diameter). Considering that quartz was the only constituent with a density of 2.65 g cm^−3^, the porosity of the compacted sand for these cells was calculated at 0.42 ± 0.03.

The porosity of the manually compacted powder was more accurately measured with mercury intrusion porosimetry (MIP) and computed microtomography (µ-CT). MIP was performed at the Federal Institute of Material Research and Testing (BAM, Berlin, Germany) with an Autopore III apparatus (Micromeritics, USA), following a modified approach after Minagawa and coworkers [45]. Two samples were prepared in glass tubes 10 mm long with 7 mm inner diameter, manually compacted in the same manner as the diffusion cells, and closed by two glass frits of 200 µm average pore size (ROBU glass filter, porosity 0, Hattert, Germany).

CT measurements were performed using a CT-alpha 160 X-ray device, (Procon, Germany100 keV X-ray energy at 125 µA tube current, with a 1 mm Al-filter on a diamond-coated target using ten 2 s exposure times on a Hamamatsu flat panel detector) directly on the same column used later for this experiment. The 800 projections obtained for each of the 8 samples (1 with a precipitation front, and 7 without) were reconstructed using the Octopus code (Inside Matters, Ghent, Belgium) in stacks of 2,048 images of 2,048 × 2,048 pixels (2,048^3^ voxels), oriented perpendicular to the diffusion direction. The reconstructed 3D volumes were segmented with image treatment software supplied by Avizo (FEI Visualization Sciences Group, Bordeaux, France). The segmentation process identifies and labels each component of an imaged system. For example, in the sea sand-celestite system, the result of the segmentation was the creation of a 3D volume with only three attenuation values (or colors): one for the pore space, one for the sand grains, and one for the celestite. For six of these samples, the segmentation was performed both with and without non-local mean filtering (noise reduction) [46]. The segmented volumes were implemented in the commercial pore-morphology model code GeoDICT (Math2Market, Kaiserslautern, Germany), which can estimate porosity, tortuosity, effective diffusivity and other petrophysical properties.

A separate imaging procedure was applied to one of the data sets (Prec1) obtained with µ-CT on the celestite precipitation front. The bright grey values for celestite, by contrast with other components of the porous material, induced artifacts during the beam hardening correction of the high resolution data set (5.89 µm voxel size). Therefore, it was pre-treated by a Matlab script to segment the dense material prior to the beam hardening correction. After the Matlab correction, a non-local mean filter was applied to the volume in 3D, and the pores and sand grains were then segmented as usual with the Avizo code.

#### Retardation of Sr by adsorption on sand

To characterize the effects of Sr^2+^ ion retardation by sorption during the celestite precipitation experiments were maintained under diffusion-controlled conditions. Sr sorption on the purified sea sand was measured in batch experiments. Aliquots of the sea-sand powder were suspended in 20 mL of a 1 or 100 mmol L^−1^ NaCl background electrolyte solution, and spiked with SrCl_2_ ranging from 1 µ L^−1^ to 0.1 mmol L^−1^. We varied the solid to liquid ratio (between 10 and 150), the contact times, and the pH of the solution. All experimental conditions are summarized in Table 2. The suspensions were 0.22 µm membrane-filtered, and the equilibrated solutions were analyzed with ICP-OES.

### Through-diffusion experiments

#### Initial transport parameters via HTO through-diffusion experiments

The initial porosity and diffusivity of the sand was determined by through-diffusion of a conservative tracer (tritiated water, HTO) in 10 replicates (diffusion cells named Trac1 to Trac10).

Neither aqueous speciation or precipitation/co-precipitation processes, and/or sorption processes are expected to affect the mobility of tritium in soil/water systems [30]. The experiments were performed in homogeneous background electrolyte concentration, so that the HTO was the only species with a concentration gradient. Background stock solutions of NaCl were prepared at 1 mmol L^−1^ or 0.5 mol L^−1^, respectively. One reservoir was filled with the spiked solution (approx. 4.20 MBq L^−1^) while the reservoir at the opposing end was filled with the non-active solution of the same ionic strength. In the case of simple diffusion (no pore space reduction), the spiked reservoir is referred to as the high concentration (HC) reservoir, and the unspiked reservoir is referred to as the low concentration (LC) reservoir. Both were sampled twice a week in order to measure the flux of tracer from the high to the low concentration reservoir. After each sampling, the solutions were renewed to maintain the boundary conditions in the HC and LC reservoirs constant. The concentration of tracer in the LC reservoir did not exceed 1 % of the amount in the HC reservoir, and the difference in concentration in the HC reservoir did not exceed 5 % between two renewals, as recommended by van Loon and coworkers [47].

**Fig. 10:**
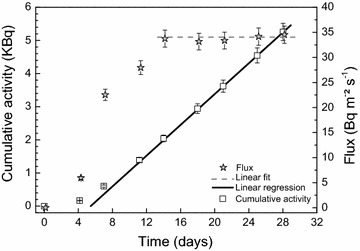
Example of a typical diffusion curve (*squares*) and flux curve (*stars*), with the linear regression of the steady state part of the diffusion curve (*full line*) and of the flux curve (*dashed line*). The linear regression (*solid line*) represents the fit of the steady state part of the cumulative activity curve (constant flux, *dashed line*). The intercept a and the slope b of the linear regression gives the porosity ε_a_ and effective diffusivity D_e_, respectively (see Eqs. 2, 3).

The initial accessible porosity *ε*_*a*_ and diffusivity *D*_*e*_ of the compacted sand columns were calculated using the obtained HTO through-diffusion curves determined in this study, following the method described in detail by van Loon et al. [47, 48]. The intercept *a* and slope *b* of the linear regression of the steady state part of the cumulative activity curves (constant flux, Fig. 10) give the porosity *ε*_*a*_ and effective diffusivity *D*_*e*_, respectively, as follows:

2 $$\varepsilon_{a} = a{ \cdot }\frac{6}{{L{ \cdot }S{ \cdot }C_{0} }}$$

3 $$D_{e} = b{ \cdot }\frac{L}{{S{ \cdot }C_{0} }}$$

where *L* is the length of the porous material (in m), *S* is the surface of the section perpendicular to the direction of diffusion (in m^2^), and *C*_0_ is the concentration of the tracer in the high concentration reservoir (in Bq m^−3^). An example of a typical through-diffusion curve, flux and linear regression is given in Fig. 10.

#### HTO through-diffusion during celestite precipitation

Celestite precipitation was induced by counter-diffusion experiments [20, 21], where the constituting cation and anion diffused from the diffusion cell reservoirs at either end of the column. The Sr-reservoir contained the cation at a concentration of 0.5 M SrCl_2_, and the opposite SO_4_-reservoir contained the anion in equal concentration of 0.5 M Na_2_SO_4_. The celestite supersaturation increased in the center of the column between the two diffusion fronts, until the salt began to precipitate once supersaturation was achieved.

Celestite (SrSO_4_) was selected as the clogging mineral phase for these experiments because of (1) its density is significantly higher than that of sand, making it possible to distinguish it from the quartz in the sand, (2) its rapid precipitation kinetics, and (3) well established knowledge concerning thermodynamic and mineralogic/crystallographic data available. The initial pH of the stock solutions in the reservoirs was 5.5, and it remained stable throughout the duration of experiments. As for simple diffusion, the Sr-reservoir was spiked with tritiated water as tracer and the reservoirs were sampled and renewed twice a week.

Three experiments were run in three different diffusion cells (Prec1, Prec2, and Prec3). The compacted sand in Prec1 was initially saturated with 1 mM NaCl, while Prec2 and Prec3 had 0.5 M NaCl. In Prec3, sodium silicate (AppliChem, Darmstadt, CAS-No.: 1344-09-8) was added to the reservoirs, in a concentration that corresponds to equilibrium with quartz (1.74 × 10^−4^ M). This was to ensure homogeneous conditions in the entire diffusion cell including the reservoirs. In Prec1 no radioactive tracer was added, as the experiment was designed to be measured with µ-CT outside the hot laboratory. The initial conditions are summarized in Table 4. All diffusion experiments were conducted with the columns oriented horizontally to eliminate any possibility of gravity-driven flow (Fig. 9).

The activity of the tracer in both reservoirs was measured by liquid scintillation counting (LSC) on a Tri-Carb 3110 TR device (Perkin Elmer, USA). Sample aliquots of 0.1–1 mL were mixed with 10 mL of LSC cocktail (Ultima Gold XR, Perkin Elmer, USA). The alpha- and beta-emission spectra were measured for 30–60 min over the 0–2,000 keV energy range.

### Post mortem analysis

The mass of celestite precipitated in the pore space of diffusion cells Prec1, Prec2 and Prec3 was quantified by dissolving the secondary phase and measuring the Sr and Si concentration released in solution. The reservoirs were removed and the compacted sand was pushed out of the column with a piston and sectioned every 5 mm. Each column provided 10 samples of about 0.6 g of material. The solid samples were rinsed with MilliQ water in a vacuum filter. Large volumes of cleaning solution were used compared with pore solution (about 10 mL of MilliQ water for about 0.2 mL of pore solution). The contact time between the samples and the cleaning solution was minimized (a few seconds) to avoid any considerable dissolution of celestite (dissolution rate of celestite at 32 °C, pH 5.7 was determined to be 10^−6.36^ mol m^−2^ s^−1^; [49]). The samples were then dried in an oven at 105 °C for at least 24 h.

The dissolution protocol used was adapted from a study on celestite dissolution kinetics [50]. The samples were suspended in 100 mL of 0.5 M HCl (pH ~2). The dissolution was performed at 70 °C for 6 h in open 250 mL Duran^®^ glass beaker using an agitator at 500 rpm. The remaining sand suspension was filtered at 0.22 µm, and two aliquots of each extract solution analyzed by ICP-OES (PerkinElmer Optima 4300 DV) for their Sr and Si content. The detection limits were 0.1 and 0.01 mg L^−1^ for Si and Sr, respectively. The remaining solid (sand matrix) after extraction was rinsed with MilliQ water and dried.

The efficiency of the method and the forms, morphology and purity of the precipitated phase were checked with SEM-EDX (FEI Quanta 650 FEG) on three solid subsamples from diffusion cells Prec1, Prec2 and Prec3 containing a celestite precipitation front before and after reaction.

### Modeling procedure

The experimental data were modeled with CRUNCHFLOW [51], using the gimrt mode and the thermoddem.dbs database [52], which applies the Debye-Hückel equation for activity corrections. For the multicomponent diffusion calculations, the diffusion coefficients in water (*D*_*w*_) at 25 °C for the main primary dissolved species were taken into account. For species with no influence on the precipitation processes, the D_w_ was set equal to that of the tracer (D_w(HTO)_ = 2.24 × 10^−9^ m^2^ s^−1^). For the other species, D_w_ was set at Cl^−^ (D_w(Cl)_ = 2.03 × 10^−9^ m^2^ s^−1^), Na^+^ (D_w(Na)_ = 1.33 × 10^−9^ m^2^ s^−1^), SO_4_^2−^ (D_w(SO4)_ = 1.07 × 10^−9^ m^2^ s^−1^), Sr^2+^ (D_w(Sr)_ = 0.794 × 10^−9^ m^2^ s^−1^), and H_4_SiO_4_ (D_w(Si)_ = 0.79 × 10^−9^ m^2^ s^−1^) as implemented in the phreeqc.dat database file. Fundamental problems arise concerning multi-component diffusion because electrolyte solutions must be electrically neutral and as a result of *D*_*w*_ differences, the flux of one ion must be linked to the flux of other ions to maintain this electro-neutrality. The complete theoretical development and general description of the so called irreversible processes of multicomponent transport is presented by Miller [53] where ion fluxes are related to electrochemical potential gradients for all ions in a system. Onsager phenomenological coefficients are defined by relating the flux of each ion to the gradients of all of the thermodynamic forces in the system [54]. The electrical cross-coupling between ions can markedly modify the diffusion process [55, 56]. This phenomenon, however, may be negligible in specific cases such as diffusion of tracers. As the required Onsager phenomenological coefficients are available for only a limited number of systems, assumptions have been made for practical simplifications. Such assumptions include use of infinite dilute ionic mobilities, negligence of off-diagonal Onsager coefficients and use of ideal chemical potential derivatives [57].

The mineral phases considered in the model were α-quartz as the porous material, and celestite as the precipitating phase. The celestite precipitation rate at 25 °C (experimental conditions 25° ± 3 °C) was set to 5.13 × 10^−8^ mol m^2^ s^−1^, and its activation energy to 8.126 kcal mol^−1^ [58]. A specific surface area of 40 m^2^ g^−1^ was used to calculate the precipitation rate for celestite. This value corresponds to an average of the values found in the literature [59–62].

A Dirichlet boundary condition (constant Sr and SO_4_ concentration in the reservoirs held throughout the experimental duration) was set. The modeling was carried out with 601 cells 0.1 mm thick in the x direction. Cells 1–50 and 551–600 correspond to the filter sets, and the last cell (601) was dedicated to the virtually forced precipitation of the HTO tracer. The experimentally determined porosity and cementation factor of simple HTO through-diffusion experiments of the sea sand material were directly implemented in the model. The temperature was set at 25 °C, and the pH was 5.5, in agreement with the experimental conditions.

## Additional file

Additional file 1. In the Supplemental Material Section results from the CrunchFlow reactive transport model sensitivity analysis concerning mesh size, tortuosity factor, porosity, cementation exponent as well as kinetics and surface area of the precipitating phase are presented.
