# Supplementary material for: Mineral precipitation-induced porosity reduction and its effect on transport parameters in diffusion-controlled porous media
Source: Geochem Trans. 2015 Sep 3;16:13. doi: 10.1186/s12932-015-0027-z (PMC4559087; doi:10.1186/s12932-015-0027-z)
Supplement: Additional file 1. — In the Supplemental Material Section results from the CrunchFlow reactive transport model sensitivity analysis concerning mesh size, tortuosity factor, porosity, cementation exponent as well as kinetics and surface area of the precipitating phase are presented. [file 12932_2015_27_MOESM1_ESM.docx]

**Appendix. Model sensitivity analysis**

The sensitivity of the CrunchFLow reactive transport model to several parameters (mesh size, tortuosity factor, porosity, cementation exponent, mesh homogeneity and heterogeneity, kinetic regime and specific surface area of the precipitating phase) was tested with and without clogging. The case study of the experiment Trac1-run1 was used, with the experimental values of porosity, tortuosity and cementation exponent obtained from the linear regression method. The diffusion cell Trac1-run1 consisted of a 50 mm long, 10 mm diameter compacted purified sea sand column, with a 2 mm thick filter support at each end of the column, for a total length of 54 mm. The reservoirs and pores contained a solution of 1 mmol L^-1^ NaCl, and the HTO tracing experiment lasted 26 days. The HTO concentration in the high concentration reservoir was of 4900 Bq mL^-1^ (4.63 × 10^-9^ mol L^-1^). The porosity, obtained with the Linear Regression (LR) method was 0.415 ± 0.021. The LR cementation exponent was equal to 2.10 ± 0.10, and the LR tortuosity factor to 0.391 ± 0.111.

**Non-reactive transport model**

The sensitivity of the model was tested on the simple HTO through diffusion, prior to porosity clogging. The configurations tested are reported in table S1. The modeled HTO fluxes are reported in fig. S1. Two different parameters can be used in the D_e_/ε relationship as described by Archie's law in the CrunchFlow code - the cementation exponent, m, or the tortuosity factor, τ:

$$\frac{D_{e}}{D_{w}}=\varepsilon^{m}=\varepsilon\cdot\tau$$

A specific tortuosity factor for each mesh-cell can be defined, which is an advantage in a medium with heterogeneous tortuosities such as ours. However, when using the cementation exponent option, only one value can be defined for the whole system. The modeled flux curves are therefore significantly different than for the tortuosity option. To correct this, it is possible to calculate a theoretical porosity for the filter supports, which would correspond to a tortuosity of 1.00 with the cementation exponent of the porous material. For the filter supports of the sand system, the corrected porosity used in the sensitivity analysis runs was 0.623. The flux thus modeled is in good agreement with the "tortuosity" model.

The model is not dependent on the mesh size. It is, however, quite sensitive to the cementation factor value: a variation in the cementation factor of about 5% leads to a 10% difference in the flux at 26 days. A similar variation of 5% in the tortuosity only yields to a 5% variation in the flux at 26 days. The tortuosity factor option was thus chosen over the cementation factor option, as it is more convenient, reliable (no need to re-calculate the porosity of the filter supports) and as the model is less sensitive to this parameter.

**Reactive transport model**

The sensitivity of the reactive transport model on the porosity, tortuosity factor and cementation exponent of the sand was tested, as for the non-reactive model previously discussed. A typical precipitation experiment was used to set-up the model. For that purpose, a 0.5 mol L^-1^ SrCl_2_ solution was introduced in the first reservoir (the one containing the tracer), and 0.5 mol L^-1^ Na_2_SO_4_ in the second reservoir. The pore solution was 0.5 mol L^-1^ NaCl.

The major difference with the non reactive model was that the tortuosity factor could not be used, as it gave irrelevant results. Therefore, the cementation exponent was used instead, with a modified porosity for the filter supports as described previously. The different configurations tested are reported in table S2. The modeled HTO fluxes are reported in fig. S2.

The porosity clogging is translated in the HTO flux by a significant drop and stabilization at low values. A steep drop is representative of a fast clogging. The reactive transport model is very sensitive to the mesh size: the smaller the mesh size, the faster the clogging. This can be explained simply by the fact that a small mesh cell will be filled with the precipitate faster than a bigger one. The HTO flux is similarly sensitive to the sand porosity and cementation exponent. A 5% variation in either one of these parameters leads to a difference in the peak value at 15 days of 10 to 12%. The effect is also pronounced in the tailing of the flux, after the drop.

In addition to the usual parameters, the sensitivity of the model on the precipitation kinetic regime and specific surface area (SSA) of the celestite was tested. For this, the model 10c was used (which was run with precipitation kinetics), and run for celestite local equilibrium (model 10c6), and with various SSA. The SSA of the celestite precipitating in the pore space is unknown for the present system. Published data vary between 0.44 and 72.7 m^2^g^-1^ (e.g. Sühnel and Handlirova, 1984; Liu et al., 2005). Two commercially available powders were measured with N_2_-BET in another study performed in the same laboratory, and were found to have a SSA of 0.5252 and 0.9981 m^2^g^-1^. The model was run with the smallest (0.44 m^2^g^-1^, model 10c7), the average (40 m^2^g^-1^, model 10c) and the biggest (72.7 m m^2^g^-1^, model 10c8) values. Results are reported in fig. S3. The model is only slightly dependent on the kinetic regime, the biggest difference between the 2 HTO flux curves being in the tailing, after the drop. The 2 curves seem to reach the same flux value around 60 days. In a similar way, the model in only slightly dependent on the celestite SSA. The curves for 40 and 72.7 m^2^g^-1^ are very similar. The curve for the 0.44 m^2^g^-1^ case is quite different, as the peak is broader: the precipitation takes about 5 additional days to influence the flux (drop). However, the minimum flux value is reached very shortly after the other cases, and the flux value reached is also very similar.

Table S1: Different configurations tested for the 1D transport model sensitivity analysis (no clogging).


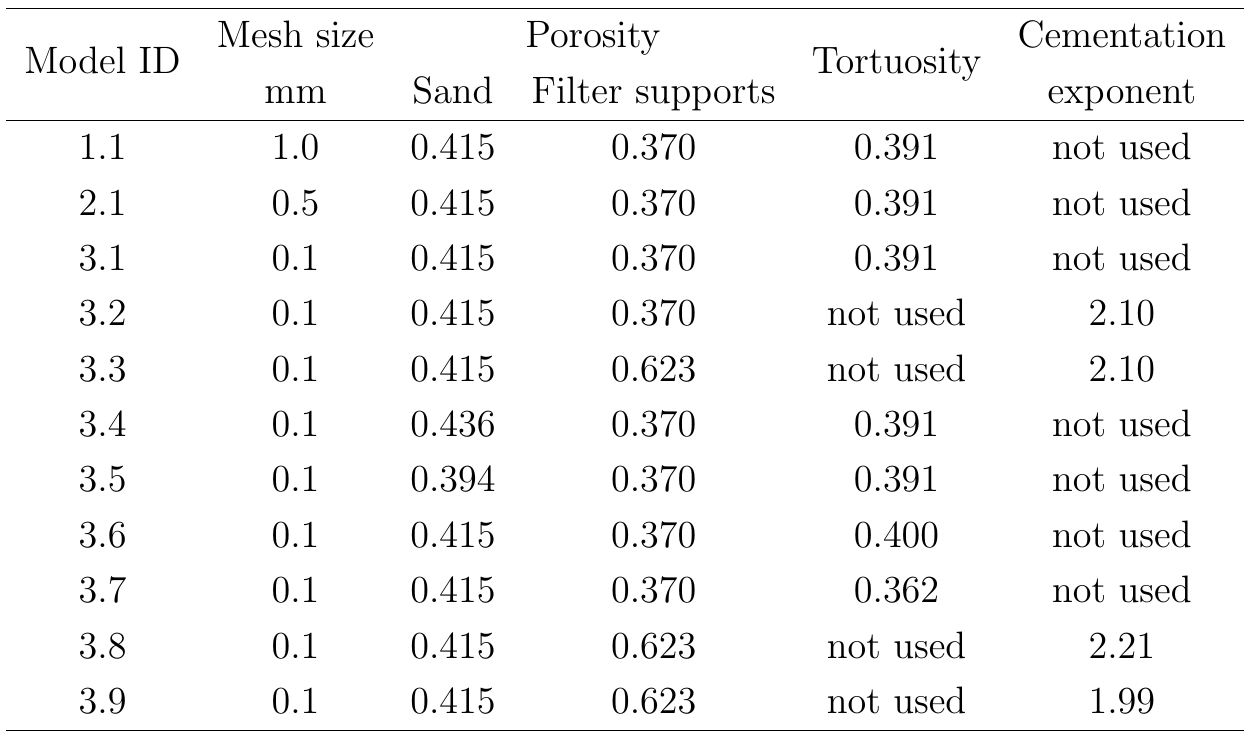


Table S2: Different configurations tested for the 1D transport model sensitivity analysis (with porosity clogging).


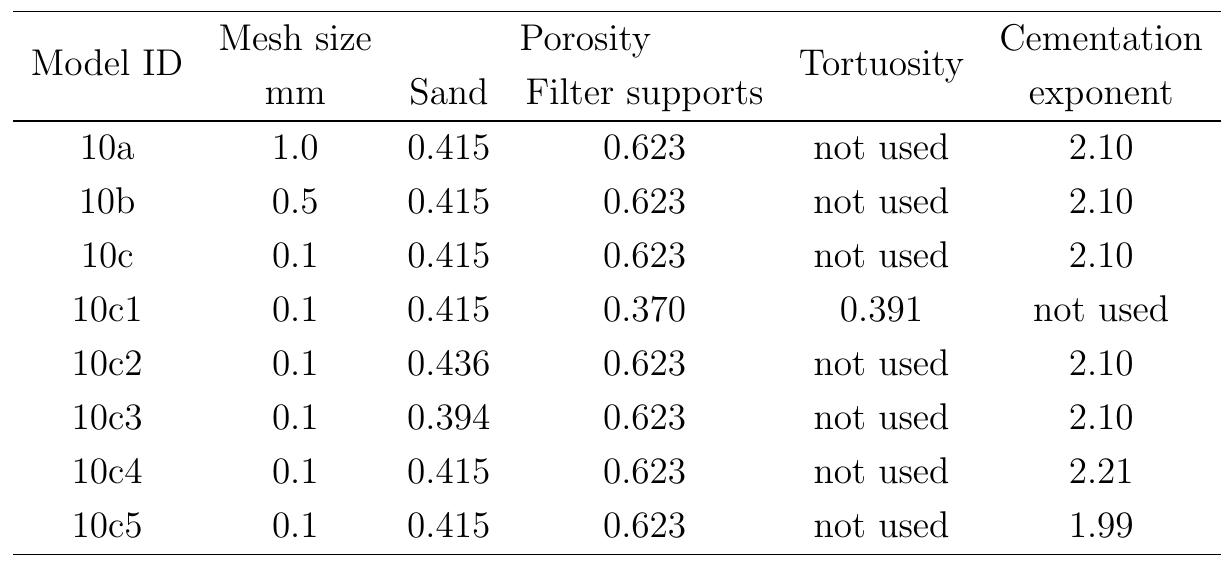


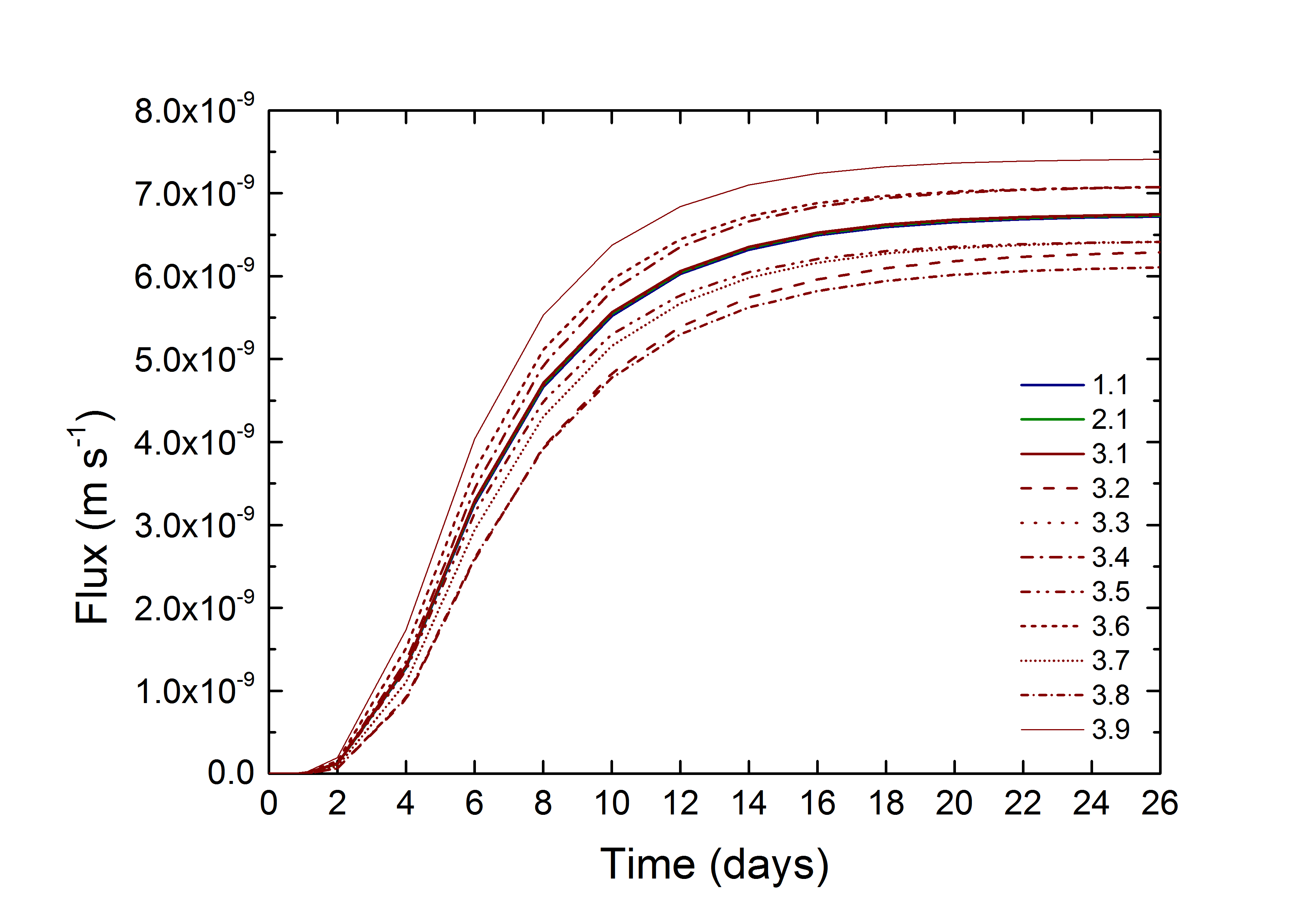


Figure S1: Modeled HTO fluxes for different configurations tested for the 1D simple transport model (no porosity clogging) sensitivity analysis.


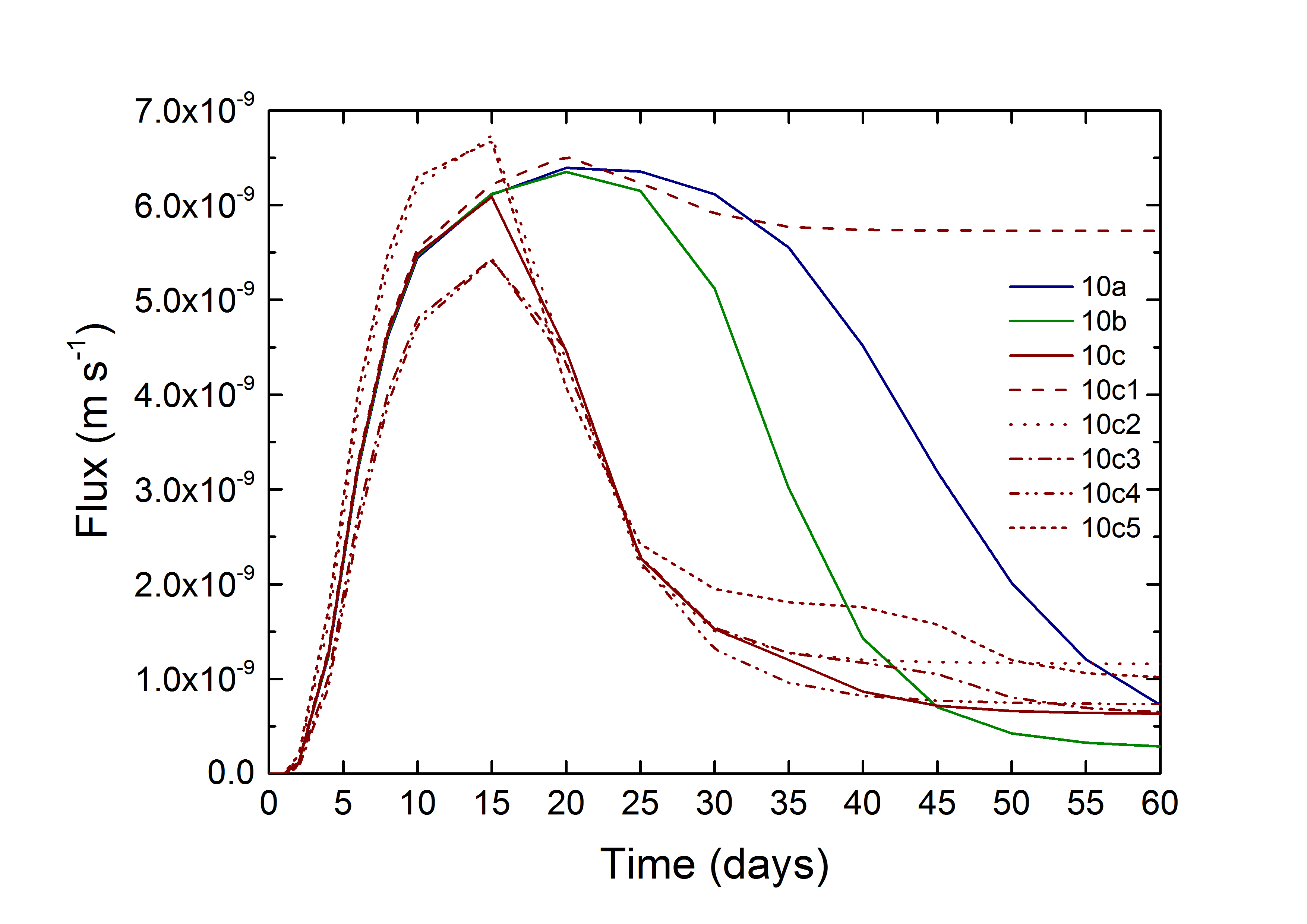


Figure S2: Modeled HTO fluxes for different configurations tested for the 1D reactive transport model (porosity clogging) sensitivity analysis.


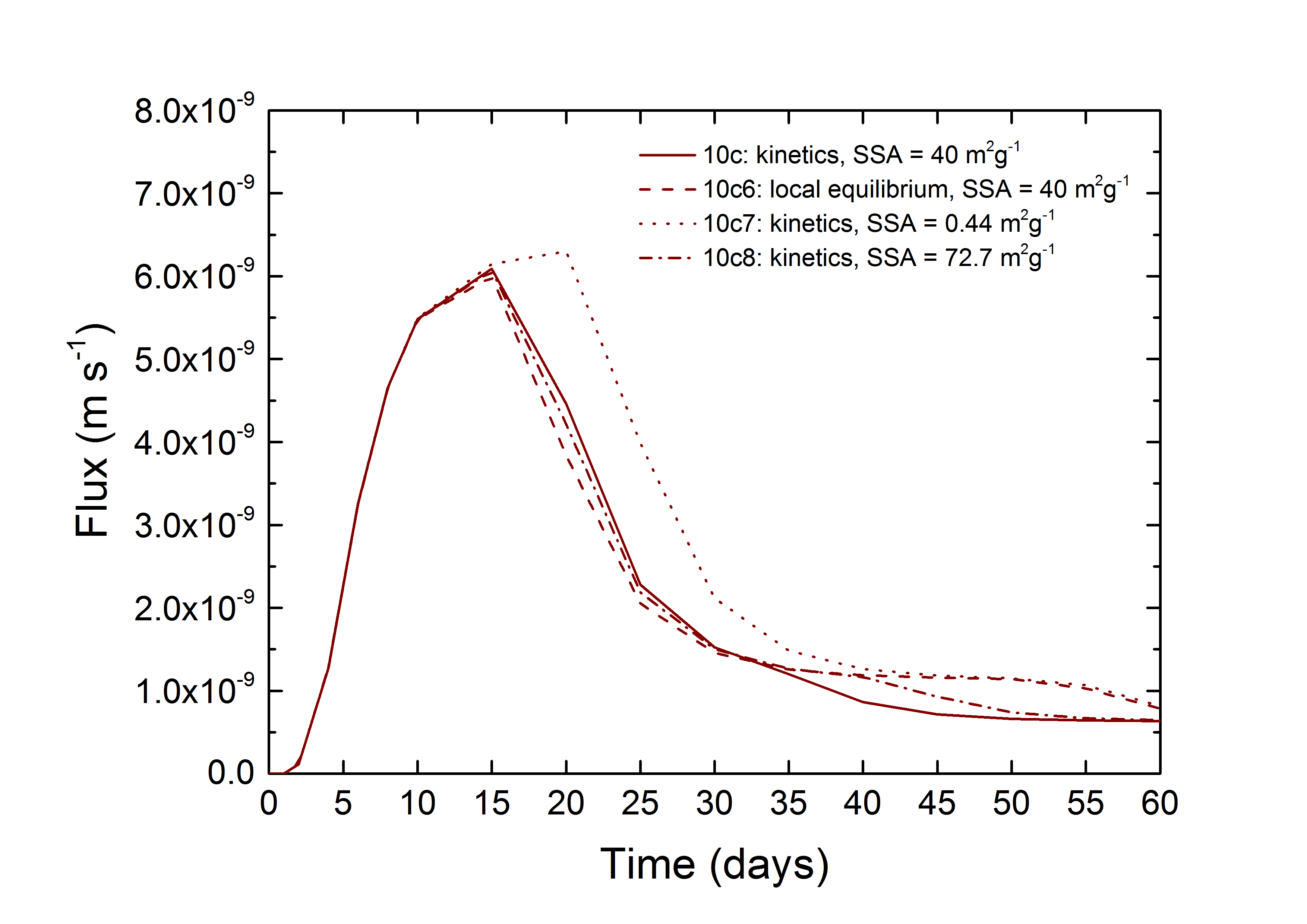


Figure S3: Modeled HTO fluxes for different configurations tested for the 1D reactive transport model (porosity clogging) sensitivity analysis to specific surface area and precipitation kinetics of celestite.
